# Preclinical Targeting of Human Acute Myeloid Leukemia Using CD4-specific Chimeric Antigen Receptor (CAR) T Cells and NK Cells

**DOI:** 10.7150/jca.28952

**Published:** 2019-07-23

**Authors:** Huda Salman, Kevin G. Pinz, Masayuki Wada, Xiao Shuai, Lulu E. Yan, Jessica C. Petrov, Yupo Ma

**Affiliations:** 1 Department of Internal Medicine, Stony Brook Medicine, Stony Brook University Medical Center, Stony Brook, NY 11794, USA; 2 iCell Gene Therapeutics LLC, Research & Development Division, Long Island High Technology Incubator, Stony Brook, NY 11790, USA; 3 Department of Hematology, West China hospital of Sichuan University, Chengdu, P.R. China

**Keywords:** NK cells, immunotherapy, T-cell malignancies, chimeric antigen receptors, AML

## Abstract

Acute myeloid leukemia (AML) is an aggressive malignancy lacking targeted therapy due to shared molecular and transcriptional circuits as well as phenotypic markers with normal hematopoietic stem cells (HSCs). Identifying leukemia specific markers expressed on AML or AML subtypes for therapeutic targeting is of exquisite clinical value. Here we show that CD4, a T lymphocytes membrane glycoprotein that interacts with major histocompatibility complex class II antigens and is also expressed in certain AML subsets but not on HSCs is a proper target for genetically engineered chimeric antigen receptor T cells (CAR-T cells). Treatment with CD4 redirected CAR-T cell (CD4CAR) specifically eliminated CD4-expressing AML cell lines *in vitro* and exhibited a potent anti-leukemic effect in a systemic AML murine model *in vivo*. We also utilized natural killers as another vehicle for CAR engineered cells and this strategy similarly and robustly eliminated CD4- expressing AML cells *in vitro* and had a potent *in vivo* anti-leukemic effect and was noted to have shorter *in vivo* persistence. Our data offer a proof of concept for immunotherapeutic targeting of CD4 as a strategy to treat CD4 expressing refractory AML as a bridge to stem cell transplant (SCT) in a first in human clinical trial.

## Introduction

Despite the unprecedented progress in understanding the genetic and molecular biology of acute myeloid leukemia (AML), this has not translated into significant advances in therapy and the improvements that have occurred are primarily the result of dose escalation of standard agents during induction and consolidation and improvements in supportive care. Patients deemed refractory to these approaches may not qualify for curative SCT because of persistent or residual disease.

The striking success of CD19-specific chimeric antigen receptor T cell (CAR-T) therapies against acute lymphoblastic leukemia has not yet been matched in AML 1. One major obstacle to targeting AML with CAR-Ts is that many myeloid antigens are expressed at similar levels on normal and malignant cells. Eliminating leukemic cells therefore may occur at the expense of normal myeloid tissue toxicity, including myeloid progenitor cells, resulting in an unacceptable “on target, off tumor” effect. Several preclinical studies have reported CARs targeting AML-associated antigens such as Lewis Y, CD33, CD44vCD123, and folate receptor β (FRβ) 2-9. Among these, Lewis Y, CD33, and CD123 have been used clinically but sustained complete responses have not yet been reported. Toxicities toward normal hematopoietic progenitor cells (HPCs) associated with the CD33 and CD123 CAR-T cell treatments have also been of particular concern.

CD4 is expressed in 65.0% and 78.3% in M4 and M5 subtypes of AML, respectively and in 30-40% of the other AML subtypes 10. CD4 is not expressed on hematopoietic stem or progenitor cells, nor is it expressed on non-hematopoietic cells 10, 11. It has been shown that CD4 depletion utilizing monoclonal antibodies in clinical trials was well-tolerated and reversible. Observed toxicities offer some insight into safety, but cannot be fully extrapolated to CAR T cells 12-15. Our group obtained an FDA IND (No 17945) to initiate a CD4CAR phase1 clinical trial in CD4 positive T cell hematological malignancies. This trial will report on the safety of CD4 redirected CAR cell therapy in humans.

While CAR-expressing T cells have entered successfully clinical trials, experience with CAR-engineered NK cells is mainly restricted to pre-clinical investigations and predominantly to NK cell lines. To date, pre-clinical data have been reported for CAR-modified primary human NK cells redirected against CD19, CD20, CD244, and HER2 as well as CAR-expressing NK-92 cells targeting a broader range of cancer antigens 16-20. Natural killer (NK) cells may represent alternative cytotoxic effectors for CAR-driven cytolysis. Allogeneic NK cells are expected to induce an immune response and be rejected after a few days, and even autologous NK cells should disappear relatively rapidly from the circulation, owing to their limited lifespan.

Here we present CD4-directed CAR T cells and CAR NK cells as potent and specific approach to eradicate CD4+ myeloid malignant cells of AML *in vitro* and in mouse xenografts. Our preclinical data set the bases for utilizing CD4-directed CAR T cells and CAR NK cells as a novel and effective treatment for patients with refractory CD4 + AML to eliminate residual disease as a bridge to more definitive therapy with allogeneic SCT.

## Materials and Methods

### Blood donors, primary leukemia cells, and cell lines

DC4 + human primary AML samples and normal peripheral blood mononuclear cells (PBMCs) were obtained from residual samples using a protocol approved by the Institutional Review Board of Stony Brook University. THP-1, U937, TALL104, and NK-92 cell lines were obtained from ATCC (Manassas, VA, USA). MOLM-13 was obtained from AddexBio (San Diego, CA, USA) T cells were cultured in filtered T cell media, defined as 50% AIM V, 40% RPMI 1640 and 10%FBS, with 1% Pen/Strep (all Gibco, Waltham, MA, USA) and supplemented with IL-2 (300 IU/mL; Peprotech, Rocky Hill, NJ, USA), unless otherwise specified. NK-92 cells were cultured in filtered NK cell media, defined as alpha-MEM without ribonucleosides and deoxyribonucleosides with 2mM L-glutamine, 1.5 g/L sodium bicarbonate (Gibco), 12.5% heat-inactivated horse serum (Gibco), 12.5% heat-inactivated FBS (Atlanta Biologicals, Atlanta GA, USA), 1% Pen/Strep (Gibco), 0.2% inositol (Sigma), 0.02% folic acid (Fisher), and 50 uM beta-mercaptoethanol (Fisher), supplemented with IL-2 (300 IU/mL), unless otherwise specified. THP-1, U937, and MOLM-13 cell lines were cultured in RPMI, 10% FBS, 1% Pen/Strep (Gibco). TALL104 cells were cultured in IMDM adding 300 IU/ml recombinant human IL-2, 2.5 mg/ml human albumin, 0.5 mg/ml D-mannitol, and 20% FBS.

### Co-Culture target cell ablation assays

In the CAR T cell co-cultures, CD4CAR T cells or GFP T cells (control) were incubated with target cells at ratios of 2:1 and 5:1 (200,000 or 500,000 effector cells to 100,000 target cells, respectively) in 1 mL T-cell culture media without IL-2 for 24h. Target cells were THP-1, U937, and MOLM-13 cell lines (acute myeloid leukemia cell lines expressing CD4), and primary bone marrow cells from two patients with AML. All target cells were pre-stained with CMTMR (Life Technologies) to distinguish them from T cells during flow analysis. As a negative control, CMTMR-stained TALL104 cells, which do not express CD4, were also incubated with CD4CAR T cells and GFP T cells in the same ratios. After 24 hours of co-culture, cells were stained with mouse anti-human CD4-APC antibody (Tonbo, San Diego, CA, USA). For dose-dependent experiments, MOLM-13 cells were co-cultured with CAR T cells at lower ratios from 0.25:1 (25,000 effector cells to 100,000 target cells) to 5:1 with a sequential titer.

In the CAR NK cell co-culture experiment, target cells were labeled with CMTMR prior to incubation with CD4CAR NK cells or GFP NK cells (control) in IL-2 free media, and all cells were labeled with mouse anti-human CD4-APC after 24h co-culture. Following this incubation, cells were washed, centrifuged, and re-suspended in 2% formalin for flow analysis.

All of the co-culture assays were performed in two independent experiments. Analysis of anti-leukemic activity was performed by comparing the residual amount of cells left in the CD4CAR T or NK cells treated samples with the GFP control cells treated samples, and data was presented as both the tumor lysis percentage and absolute cell counts. Analysis was performed using Kaluza software (Beckman Coulter, Brea, CA, USA).

### *In vivo* mouse xenogeneic model

Two sets of NSG mice (NOD.Cg-*Prkdc^scid^ Il2rg^tm1Wjl^*/SzJ) from the Jackson Laboratory (Bar Harbor, ME, USA) were used under a Stony Brook University IACUC-approved protocol. Mice were all male and between 9 and 12 weeks old. For each set, 16 mice were irradiated with a sublethal (2.0 Gy) dose of gamma irradiation and 24h later they were intravenously injected with luciferase-expressing MOLM-13 cells via tail vein in order to form a measurable systemic leukemia. They were then randomly assigned to the treatment group or control group. In the first set, mice were injected with 1.0 x 10^6^ MOLM-13 cells and then given a course of 10 x 10^6^ CD4CAR T cells (n=8) or GFP control T cells (n=8) 3 days later via tail vein injection. In the second set, NSG mice were injected with 0.5 x 10^6^ MOLM-13 cells and then treated with a first dose of 5 x 10^6^ CD4CAR NK cells (n=8) or 5 x 10^6^ GFP control NK cells (n=8) 24 hours later, and then a second dose of 5 x 10^6^ CD4CAR NK cells or control cells on Day 10. After tumor cell inoculation, mice were monitored for systemic leukemia burden on the following sequential days (day 3, 6, 11 for the first set of mice; day 3, 7, 9 for the second set of mice). Mice were injected intraperitoneally (IP) with 100 μL RediJect D-Luciferin (Perkin Elmer, Waltham, MA, USA) and subjected to IVIS imaging (PerkinElmer). Images were analyzed using Caliper Life Sciences software (PerkinElmer), and data was analyzed as previously described (Pinz 2015, Chen 2016).

Lentivirus production, CD4CAR transduction, and validation of C4CAR expression in both NK-92 and T cells, as well as statistical analysis are described in detail in Supplementary Information.

## Results

### Generation of CD4CAR T cells

**Figure 1:**
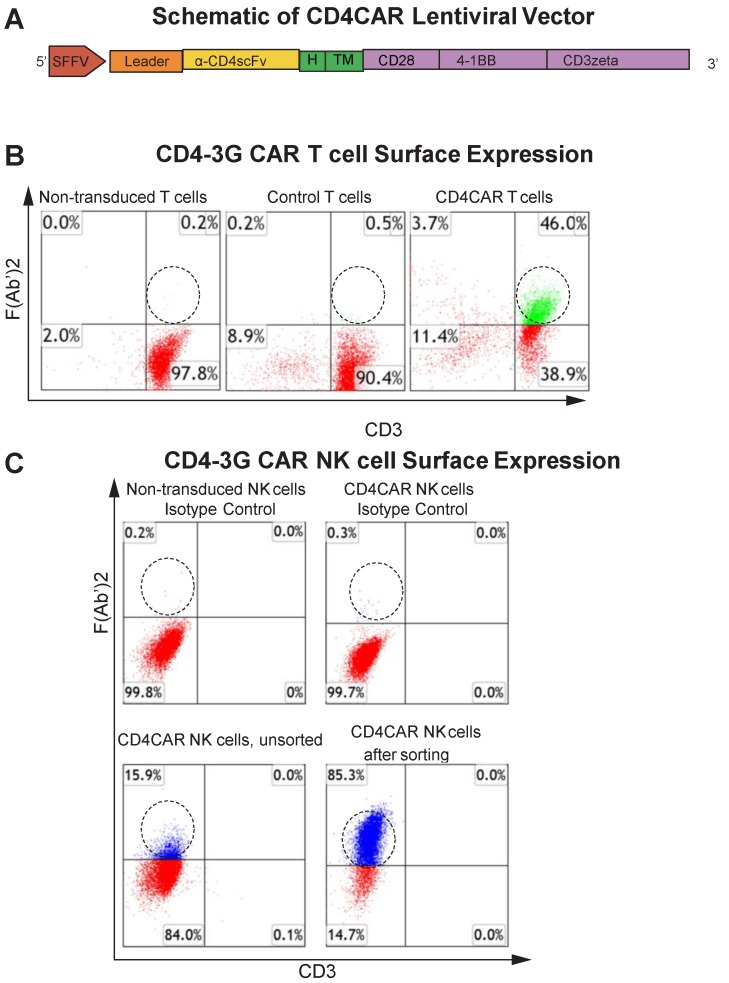
** CD4CAR construct.** (A) Schematic representation of the CD4CAR lentiviral vector (top). The CD4CAR construct is a tandem signaling domain that contains: a leader sequence; an anti-CD4scFv; a hinge domain (H); a transmembrane domain (TM); two co-stimulatory domains (CD28 and 4-1BB) that define the construct as a “third generation” CAR; and a CD3zeta intracellular signaling domain. (B) Flow cytometry analysis of CD4CAR expression on T-cell surface of non-transduced (left), GFP control (middle), and CD4CAR T-cells (right). Population in green delineates transduced CD4CAR T-cells. Gating was based off the vector and isotype controls.(C) Flow cytometry analysis of CD4CAR expression on NK-92 cell surface for non-transduced NK isotype control cells (upper left), CD4CAR NK isotype control cells (upper right), unsorted CD4CAR NK cells (lower left), and CD4CAR NK cells after sorting and expansion (lower right). Population in blue delineates transduced CD4CAR NK-92 cells. Gating was based off the vector and isotype controls.

The CD4-specific CAR (pRSC.SFFV.CD4.3G) was designed to contain an anti-CD4 scFv, a CD8-derived hinge (H) and transmembrane (TM) domains, as well as intracellular CD28 and 4-1BB domains in tandem with the CD3zeta domain, defining the construct as a third-generation CAR (Figure 1A) (Pinz, 2015). Flow cytometry analysis showed that ~46% of T cells expressed the CD4CAR F (Ab') 2 fragment after transduction (Figure 1B).

### CD4CAR T cells specifically eliminate CD4-expressing AML cell lines

**Figure 2:**
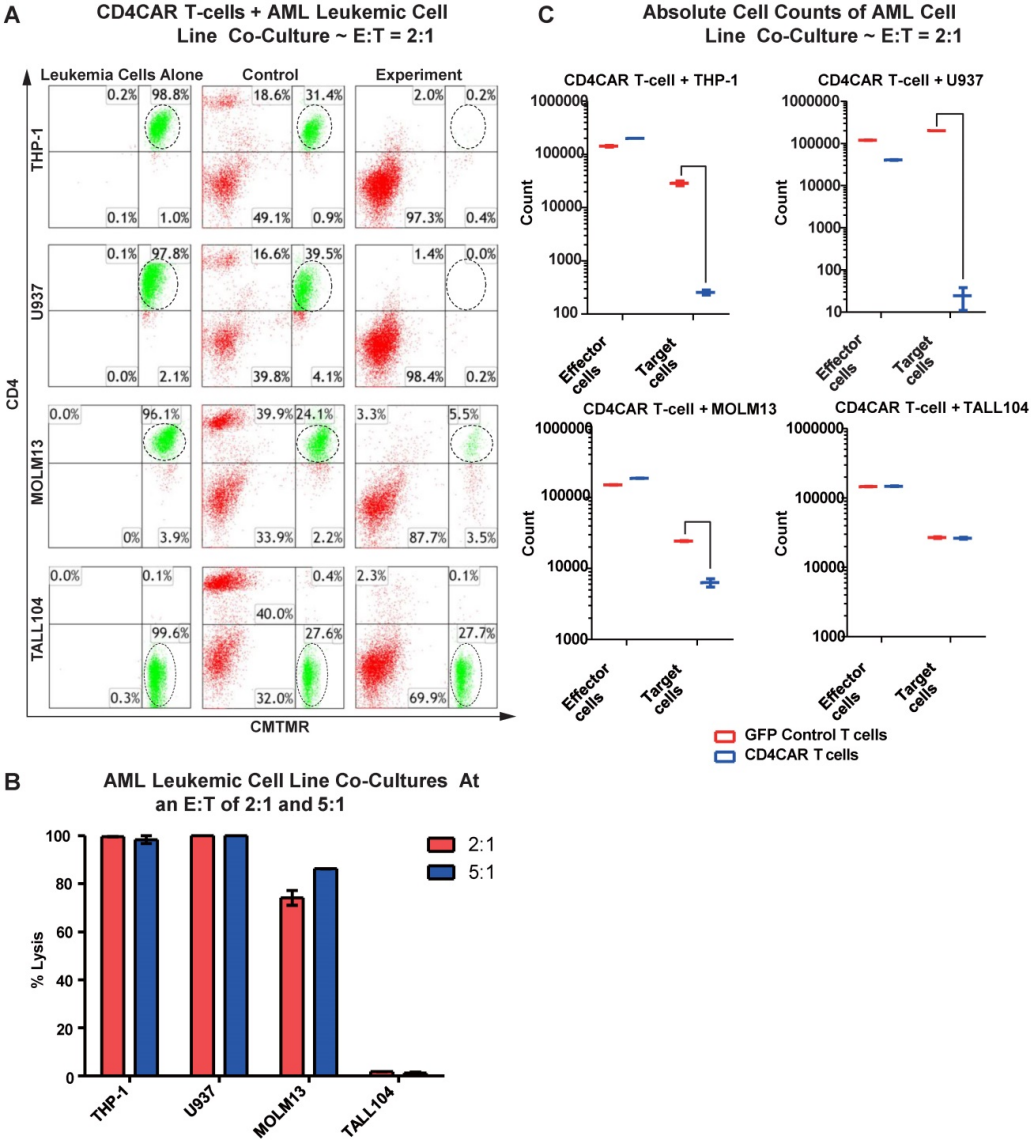
** CD4CAR T-cells ablate CD4^+^ AML cell lines *in vitro*.** (A) Co-culture experiments with CD4CAR T-cells were performed at E: T ratios of 2:1 and 5:1 for 24 hours. CD4+ target cell lines used are THP-1, U937, and MOLM13. TALL104 is a CD4- control. Target populations were quantified via flow cytometry using CD3 and pre-labeled CMTMR target cells to distinguish T-cell and target cell populations. Populations encircled highlight target cell lysis. (B) Graphical summary of CD4CAR T-cell *in vitro* assays against AML cell lines. Each bar represents the average percent cell lysis for duplicate samples; N = 2 for all.(C) Absolute cell counts of target cell cultures with CD4CAR T-cells at an E: T ratio of 2:1. Control and CD4CAR treatment samples are labeled in red and blue respectively with effector and target cell counts performed via FACS analysis.

We first evaluated the anti-leukemia activity of CD4CAR T cells in individual co-culture killing assays with three CD4-positive acute myeloid leukemia cell lines: THP-1, U937, and MOLM-13. A CD4-negative T-ALL104 cell line was used as a negative control (Figure 2A). CD4CAR T cells were tested via 24hour co-cultures using the four cell lines with effector: target (E:T) cell ratios of 2:1 and 5:1. Compared to GFP control T cells, we observed that CD4CAR T cells specifically ablated CD4-positive populations (Figure 2A). At an E:T cell ratio of 2:1, we found that CD4CAR T cells already lysed over 74% of the MOLM-13 cells, and eliminated nearly 100% of the THP-1 and U937 cells. With an E: T ratio increase to 5:1, the lysis rate increased to 86% in the MOLM13 cells, remained nearly 100% in the THP-1 and U937 cell lines. Conversely, CD4CAR T cells did not lyse the negative control T-ALL104 cells at either E: T ratio (Figure 2B).

In order to rule out the possibility that T cell expansion after antigen stimulation might confound the cytolysis rate, we also compared absolute cell counts to verify the CAR T cells cytotoxicity assay. We found that the absolute cell numbers of leukemic target cells THP-1, U937, and MOLM13 were significantly decreased in the CAR T cell treated group, compared with those in the GFP control group in the co-culture studies (Figure 2C).

Furthermore, we investigated the potential dose-dependent relationship of CD4CAR T cells by co-culturing MOLM-13 cells with sequential E: T dilution ratios of 0.25:1, 0.5:1, 1:1, 2:1, and 5:1. The result showed that target cell lysis rates were 1.3%, 20%, 64.8%, 73%, and 81%, respectively (Figure S1), therefore demonstrating a strong anti-leukemia dose-dependent effect of CD4CAR T cells.

### CD4CAR T cells specifically target and lyse CD4^+^ primary AML leukemic cells

**Figure 3:**
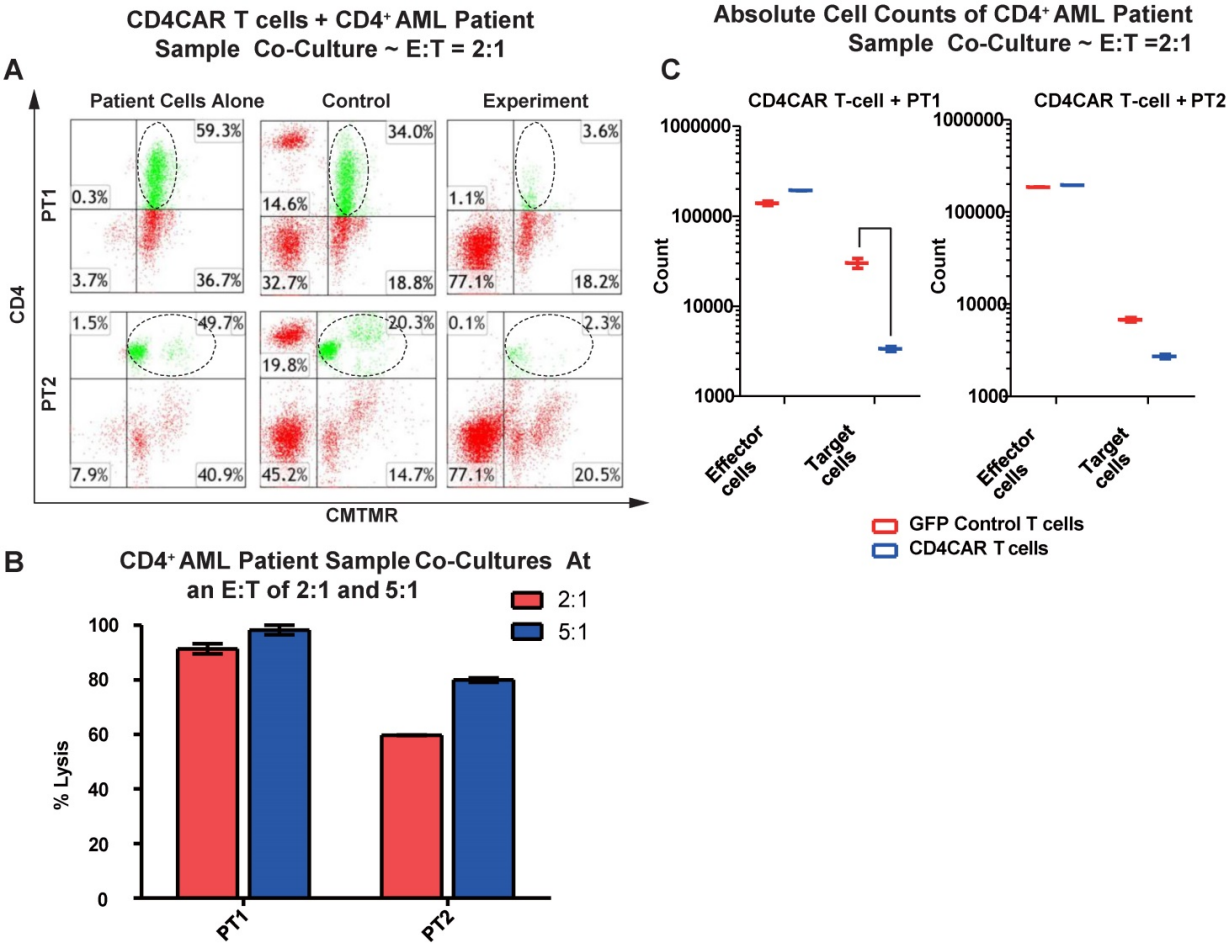
** CD4CAR T-cells ablate CD4+ AML patient cells *in vitro.***(A) Co-culture experiments with CD4CAR T-cells were performed at E: T ratios of 2:1 and 5:1 for 24 hours. Target cells used are derived from Patient 1 (PT1) and Patient 2 (PT2). Target populations quantified with flow cytometry using CD3 and pre-labeled CMTMR target cells to distinguish T-cell and target cell populations. Populations encircled highlight target cell lysis. (B) Graphical summary of CD4CAR T-cell *in vitro* assays against AML patient cells. Each bar represents the average percent cell lysis for duplicate samples; N = 2 for all. (C) Absolute cell counts of target cell cultures with CD4CAR T-cells at an E: T ratio of 2:1. Control and CD4CAR treatment samples are labeled in red and blue respectively with effector and target cell counts performed via FACS analysis.

We next tested the efficiency of CD4CAR T cells in recognizing and killing primary AML leukemic cells. Co-culture experiments were conducted using clinical samples from two patients with CD4+ acute myeloid leukemia (PT1 and PT2). The two patients' bone marrow samples contained 59.3% and 49.7% of CD4-positive cells, respectively (Figure 3A). This corresponded with the blast population in the two patients' marrows (data not shown). In each case, a high level of target cell lysis was observed at either E: T ratio, as compared to GFP control T cells (Figure 3B). The potent cell lysis activity was also confirmed by absolute cell counting, with a significant reduction in residual target cells in the CD4CAR T cell-treated group (Figure 3C), compared to the GFP control group. The results showed that CD4CAR T cells specifically targeted and eradicated primary myelogenous leukemic cells expressing CD4 *in vitro*.

### CD4CAR T cells exhibit potent anti-leukemic effects in a systemic AML mouse model *in vivo*

**Figure 4:**
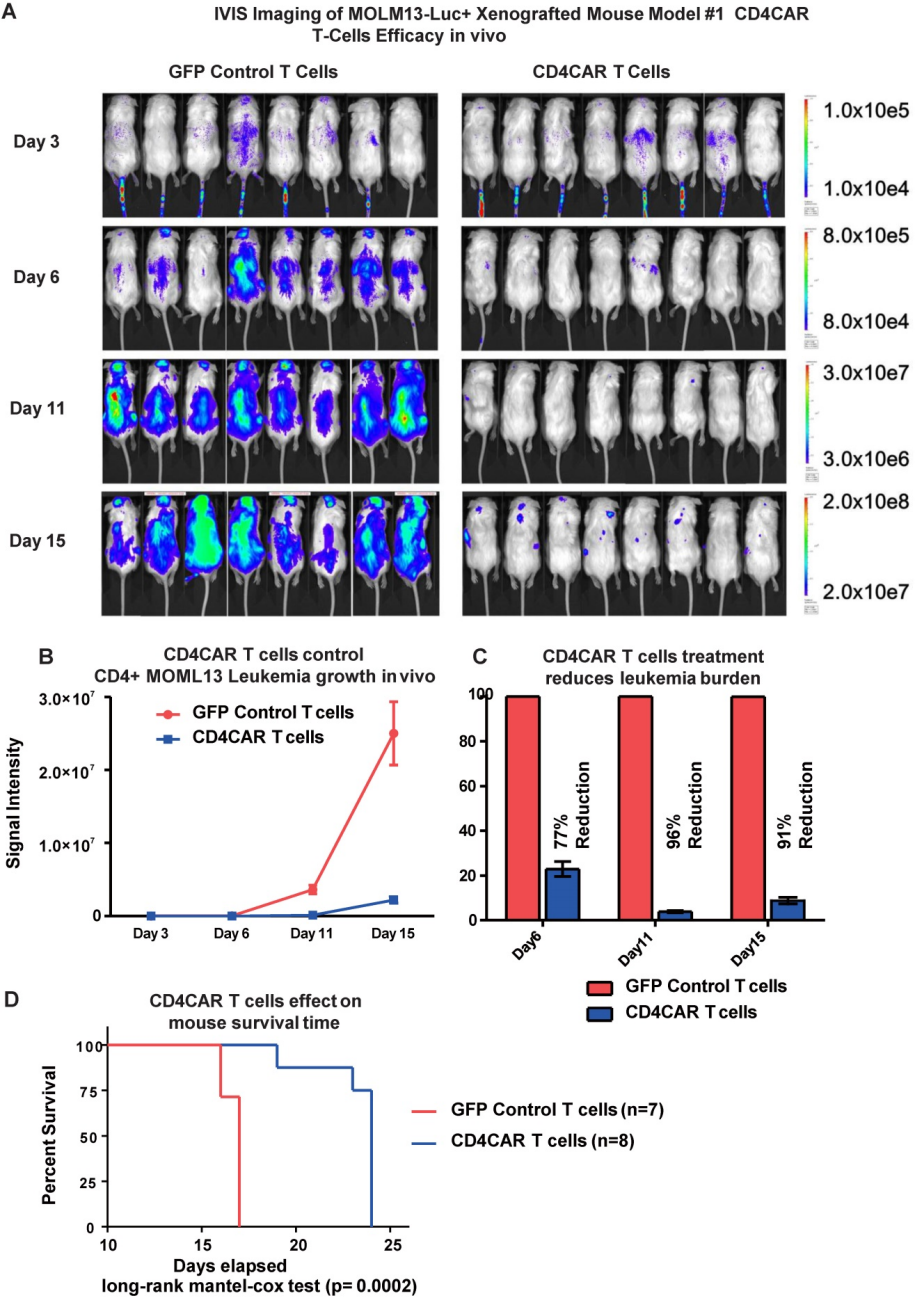
** CD4CAR T-cells demonstrate profound anti-leukemic effects *in vivo*.** (A) Elimination of luciferase-expressing MOLM-13 cells in xenografted mice treated with CD4CAR T-cells as measured via IVIS imaging. Average flux of IVIS imaging was measured and compared in the CD4CAR T-cell treated mice (right, N=8) versus that in the control T-cells treated mice (left, N=8) on day 3, 6, and 11. The percent cell lysis by CD4CAR T-cells relative to control was determined via luciferin signal. (B) CD4CAR T-cells control MOLM-13 tumor growth *in vivo*. Average light intensity (in photons per second) measured for the CD4CAR T-cell injected mice was compared to that of GFP control T-cell injected mice. P-values are indicated at specific time-points, demonstrating a statistically significant reduction in the relative tumor burden by CD4CAR T-cells as compared to GFP control on days 6 (p = .00174) and 11 (p = < .0001). (C) CD4CAR T-cells reduce MOLM-13 tumor burden *in vivo*. Percent luciferin signal measured for CD4CAR T-cell injected mice and demonstrated as percent difference in signal from GFP control T-cell injected mice. The percent reduction of tumor burden on days 6 and 11 is shown. (D) CD4CAR T-cell treated mice survive significantly longer than control mice. Kaplan-Meier survival curve for CD4CAR T-cell treated mice compared to GFP control treated mice (log-rank (Mantel-Cox) test p-value = .0002). On Day 24, all CD4CAR-treated mice were sacrificed for persistency studies.

To investigate the anti-leukemic activity of CD4CAR T cells *in vivo*, we established a xenogeneic mouse model bearing the leukemic MOLM-13 cell line. Mice were given a single dose of CD4CAR T cells or GFP control T cells and tumor burden was measured on day 3, 6, and 11 (Figure 4A, 4B). By day 6, CD4CAR T cells exhibited profound leukemia suppression, indicated as a 77% reduction of leukemic burden in the CAR T cell treated group, compared with the GFP control T cell treated group. This capacity of CD4CAR T cells to control leukemia progression was enhanced by day 11, as the reduction rate reached 96% compared with the GFP control T cell group (Figure 4C). Furthermore, the survival time of CD4CAR T cell treated mice was significantly extended compared to that of GFP control T cell treated mice (P=0.0002, Figure 4D), which indicated an efficient repression of the leukemia by CD4CAR T cells *in vivo*.

### Generation and enrichment of CD4CAR NK cells

In this study, we also developed CD4CAR NK cells to target AML. CD4CAR NK cells were transduced as previously described 22, 23, with a transduction efficiency of 16% as determined by flow cytometry. In order to further enrich for CD4CAR-positive NK cells, NK cells which expressed high levels of the construct were selected for by fluorescence activated cell sorting (FACS). The enriched CD4CAR NK cells were expanded* in vitro* and exhibited a stable expression rate over 85% (Figure 1C).

### CD4CAR NK cells robustly eliminate CD4-expressing AML cell lines

**Figure 5:**
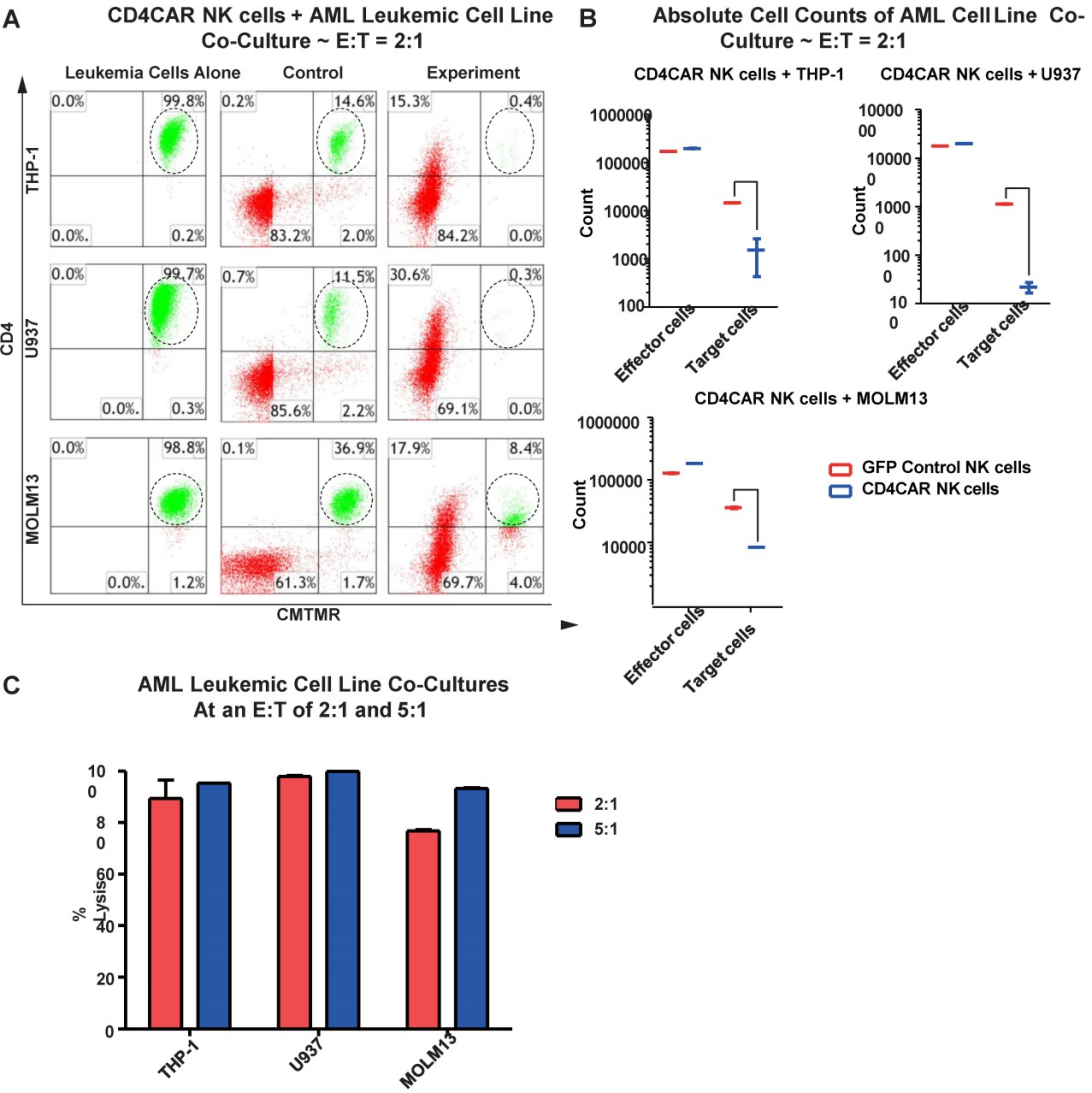
** CD4CAR NK-92 cells ablate CD4^+^ AML cell lines *in vitro*.** (A) Co-culture experiments with CD4CAR NK-92 cells were performed at E: T ratios of 2:1 and 5:1 for 24 hours. CD4+ target cell lines used are THP-1, U937, and MOLM13. Target populations were quantified with flow cytometry using CD4 and pre-labeled CMTMR target cells to distinguish NK and target cell populations. Populations encircled highlight target cell lysis. (B) Graphical summary of CD4CAR NK-92 cell *in vitro* assays against AML cell lines. Each bar represents the average percent cell lysis for duplicate samples; N = 2 for all. (C) Absolute cell counts of target cell cultures with CD4CAR NK-92 cells at an E: T ratio of 2:1. Control and CD4CAR treatment samples are labeled in red and blue respectively with effector and target cell counts performed via FACS analysis.

We next evaluated the anti-leukemic activity of CD4CAR NK cells using CD4-positive AML cell lines: THP-1, U937, and MOLM-13. We co-cultured THP-1, U937, or MOLM-13 cells with CD4CAR NK cells for 24 hours at E: T ratios of 2:1 and 5:1 (Figure 5A). Compared with GFP control NK cells, we observed that CD4CAR NK cells consistently and robustly eliminated all CD4-positive leukemic cells (Figure 5A, 5B). At an E:T ratio of 2:1, we found that that CD4CAR NK cells already demonstrated significant cytotoxicity, and were able to lyse 89%, 98%, and 77% of tumor cells in co-culture with THP-1, U937, and MOLM-13 cells, respectively. When the E: T ratio increased to 5:1, nearly 100% lysis was observed in all three cell lines (Figure 5C).

### CD4CAR NK cells specifically target and lyse CD4+ primary AML leukemic cells

**Figure 6:**
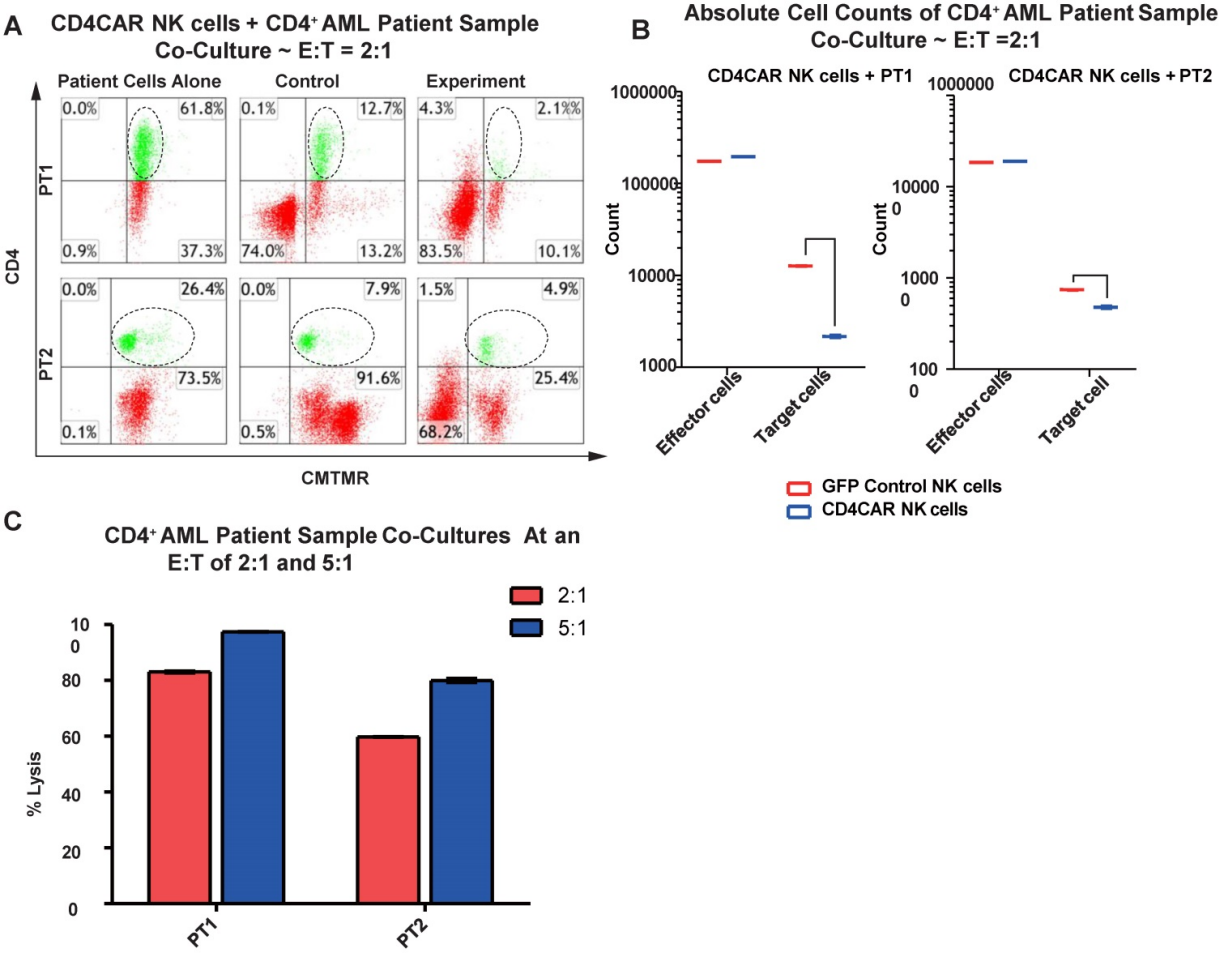
** CD4CAR NK-92 cells ablate CD4^+^ AML patient cells *in vitro*.** (A) Co-culture experiments with CD4CAR NK-92 cells were performed at an effector to target ratio of 2:1 and 5:1 for 24 hours. Target cells used are derived from Patient 1 (PT1) and Patient 2 (PT2). Target populations were quantified with flow cytometry using CD4 and pre-labeled CMTMR target cells to distinguish NK and target cell populations. Populations encircled highlight target cell lysis. (B) Graphical summary of CD4CAR NK-92 cell *in vitro* assays against AML patient cells. Each bar represents the average percent cell lysis for duplicate samples; N = 2 for all. (C) Absolute cell counts of target cell cultures with CD4CAR NK-92 cells at E: T ratios of 2:1. Control and CD4CAR treatment samples are labeled in red and blue respectively with effector and target cell counts performed via FACS analysis.

**Figure 7:**
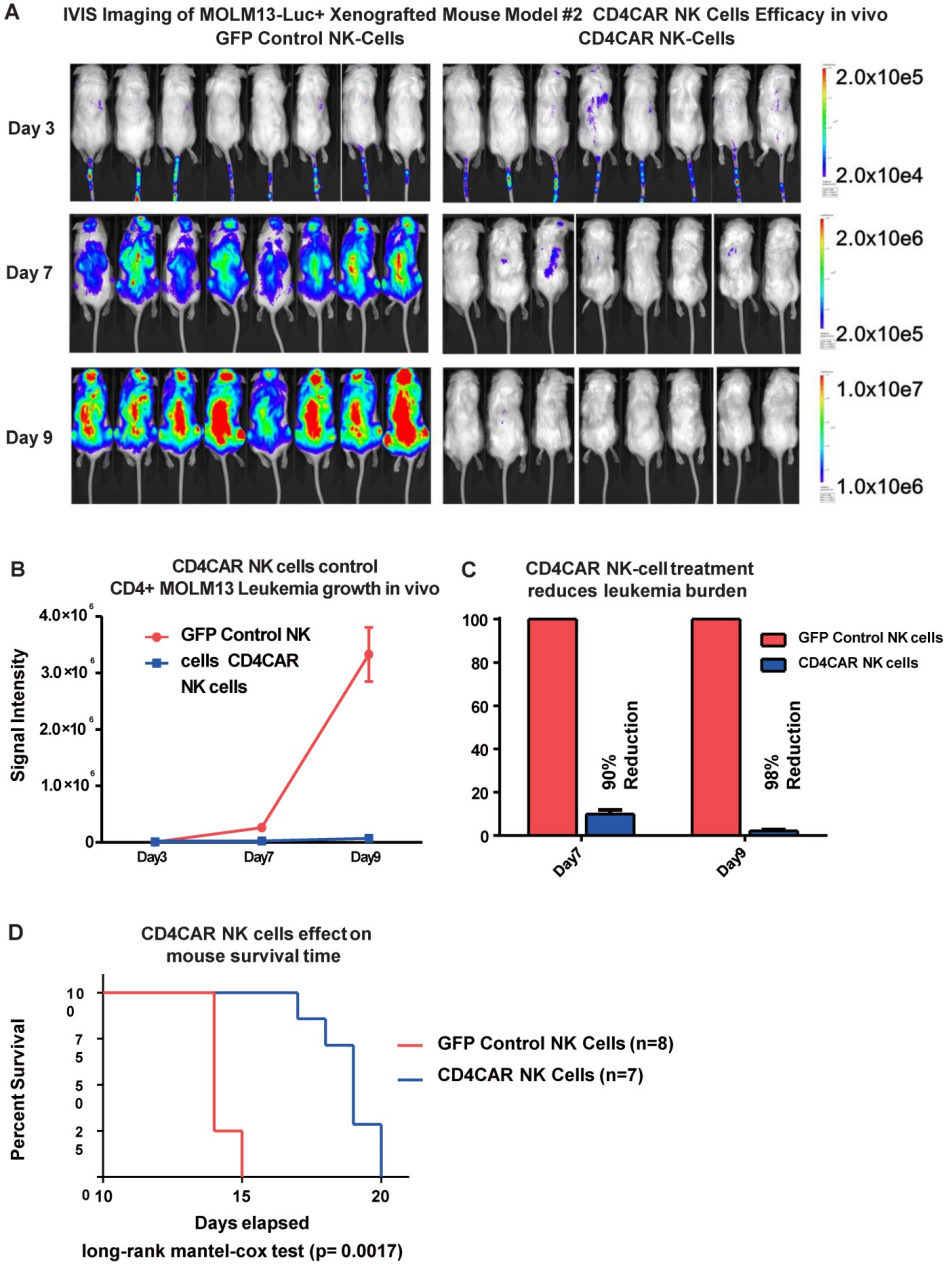
** CD4CAR NK-92 cells demonstrate profound anti-leukemic effects *in vivo*.** (A) Elimination of luciferase-expressing MOLM-13 cells in xenografted mice treated with CD4CAR NK-cells as measured via IVIS imaging. Average flux of IVIS imaging was measured and compared in the CD4CAR NK-cell treated mice (right, N=8) versus that in the control NK cells treated mice (left, N=8) on days 3, 7, and 9. The % cell lysis by CD4CAR T-cells relative to control was determined via luciferin signal. (B) CD4CAR NK-cells control MOLM-13 tumor growth *in vivo*. Average light intensity (in photons per second) measured for the CD4CAR NK cell injected mice was compared to that of GFP control NK-cell injected mice. P-values are indicated at specific time-points, demonstrating a statistically significant reduction in the relative tumor burden by CD4CAR NK-92 cells as compared to GFP control on days 7 (p = <.00001) and 9 (p = <.00001). (C) CD4CAR NK cells reduce MOLM-13 tumor burden *in vivo*. Percent luciferin signal was measured for CD4CAR T-cell injected mice and demonstrated as percent difference in signal from GFP control T-cell injected mice. The percent reduction of tumor burden on days 7 and 9 is shown. (D) CD4CAR NK cell treated mice survive significantly longer than control mice. Kaplan-Meier survival curve for CD4CAR NK cell treated mice compared to GFP control treated mice (log-rank (Mantel-Cox) test p-values = 0.0017). On Day 24, all CD4CAR-treated mice were sacrificed for persistency studies.

We then verified the efficiency of CD4CAR NK cells in specifically recognizing and eliminating CD4-positive primary leukemic cells from AML patient bone marrow samples. Again, two patient samples PT1 and PT2 were co-cultured with CD4CAR NK cells at E: T ratios of 2:1 and 5:1 (Figure 7A). A robust lysis rate was already observed at E: T ratio of 2:1, and the lysis rate further approached 80-100% when the E: T ratio increased to 5:1 (Figure 6A, 6C). The result of absolute cell counts also confirmed a significant decrease of the target leukemic cells after co-culture with CD4CAR NK cells (Figure 6B).

### CD4CAR NK cells exhibit potent anti-leukemic effects in a systemic AML mouse model *in vivo*

To further model *in vivo* activity, we examined the anti-leukemic effects of CD4CAR NK cells in a MOLM-13 leukemic mouse model. Mice were treated with either CD4CAR NK cells or GFP control NK cells and tumor burden was measured on days 3, 7, and 9 (Figure 7A). CD4CAR NK cells efficiently suppressed leukemic growth, with a 90% tumor reduction by day 7 compared with control NK cells. This difference increased to 98% by day 9 (Figure 7B, 7C). To maintain the NK cell population, a second treatment dose was given on day 10. This resulted in a significantly prolonged survival time in CAR NK cell treated mice than in control NK cell treated mice (P=0.0017, Figure 7D).

## Discussion

The “on-target off-tumor” myelotoxicity remains a challenge in carrying over the success of targeted therapy from lymphoid leukemias to myeloid targeting because myeloid antigens are likely shared on the hematopoietic stem cells 2, 3, 5, 6. We previously showed that our CD4CAR did not affect the CD34+ cord blood granulocyte/macrophage or erythroid colony formation (CFU) *ex vivo*, which indicates that HSC ability to populate the marrow is not affected by CD4CAR treatment 10. Our novel approach using CD4-directed therapy for AML is an alternative way to target an antigen that is not ubiquitously expressed on hematopoietic stem cell or non-hematopoietic cells, therefore circumventing potential relevant toxicities. The use of CD4, traditionally thought of as a lymphoid marker, to target a myeloid malignancy represents an innovative therapy that can supplement current AML available treatments. We used two effector systems, T-cells and NK cells, to implement CD4CAR therapy, creating novel treatment options for relapsed and refractory patients where none previously existed. We showed that CD4CAR-engineered T cells and NK cells specifically killed CD4+ malignant AML cell lines and primary AML patients' blasts *in vitro*. Furthermore, these CD4CAR-redirected effector cells exhibited profound killing capacity against CD4-positive AML cells *in vivo*, and efficiently controlled leukemia progression and prolonged survival in xenograft mouse models.

CD4 is expressed on a significant number of AML patients. 65.0% and 78.3% of M4 and M5 subtypes, respectively and in 30-40% of the other AML subtypes express this antigen on blasts cells 10. In our experience, when positive, CD4 expression corresponded with the blast percentage (data not shown). CD4CAR T cell therapy for CD4+ AML has the potential to reduce leukemic burden or to induce remission in refractory cases in preparation and as a bridge to definitive hematopoietic stem cell transplantation. Conversely, shorter lived CAR NK cells last only around 2 weeks, enabling a “hit and run” approach 24 and the potential to accomplish both of these treatment goals. NK cells also offer additional CAR-independent, granule-mediated killing strategies via antibody-dependent cell-mediated cytotoxicity (ADCC) and death receptor pathways 25, 26. CAR NK cells also function as “serial killers”, destroying multiple target cells in succession 27. Together, these properties of CAR NK cells make them particularly suited for a rapid, yet transient reduction in tumor burden. Moreover, CAR NK cells pose little or even no risk of graft-versus-host disease (GvHD) reaction, and do not release cytokine-storm inducing IL-6, the greatest risk in CAR T cell therapy 28. For patients who are unfit for more intensive therapy using CAR T cell therapy, the shorter lifespan and reduced toxicity of CAR NK cells may serve as a palliative treatment and eliminate concerns of long-term side effects as seen in CAR T cells and potentially qualifying these patients for curative SCT utilizing reduced conditioning. Additionally and because of their short life NK CAR cells might potentially be given more than once if needed.

Our studies found that CD4CAR-redirected immune effector cells efficiently control leukemia progress *in vitro* and *in vivo*. Irrespective of a CD4-depleted environment, CD8+ T cells alone are likely enough to eliminate tumors. A recent report showed that infusion of CD8+ CAR-modified T cells was sufficient to maintain long-term target cell eradication and leukemia remission in a mouse model 29. Recent studies also showed that memory CD8+ cells or less-differentiated cell subsets were associated with superior T cell engraftment, persistence, and antitumor immunity 30,31. Notably, our CD4CAR T cells had a predominant cell population with a central memory-like phenotype (CD8+CD45RO+CD62L+) at the end of each culture, as reported in our previous study. Additionally, CD4CAR therapy might provide an added benefit of depleting CD4+ T cells and modulating the tumor microenvironment. CD4-positive cells include Tregs, Th2 cells, Tr1/3 cells, a subpopulation of myeloid-derived suppressor cells (MDSCs), and dendritic cells (DCs) 33, 34, 35. Recent studies have shown that increased frequency of Treg cell number correlates to cancer progression, and that Tregs are important immunosuppressive factors in the AML microenvironmen 36, 37. Tregs limit CD8+ effector cell differentiation and NK cell cytotoxicity (Pedroza-Pacheco 2013) 38 and impair the efficacy of adoptive T-cell therapy 39.

While transient CD4+ lymphodepletion remains a valid concern together with the “on-target off-tumor” toxicities associated with immunotherapy. We envision that treatment with the CD4CAR will be most helpful and best applied to reduce tumor burden or induce remission in preparation “as a bridge” to more definitive therapy with hematopoietic stem cell transplantation. This approach will further minimize long term lymphodepletion via elimination of the CAR cells by pre-transplant conditioning regimens and consequently via a whole new allogeneic lymphoid engraftment. Utilization of CD4CAR in these otherwise refractory patients will provide a potentially curative treatment if they successfully qualify for SCT and again, without significant concerns about long term effects of CAR persistence and associated lymphodepletion. As a further precaution against off-target cytotoxicities and CD4+ lymphodepletion especially for those patients who cannot make it to transplant, alemtuzumab-based safety switch is expected to allow fast and thorough pharmacologic ablation of these cells. Alemtuzumab could eliminate not only peripheral blood CAR T cells, but also tissue-infiltrating CAR T cells making CD4CAR safe to develop for clinical use, supplementary FIG 2S. Furthermore, in this study we also present the option of utilizing NK CAR that is typically associated with production of a different spectrum of cytokines compared to T cells including γ-Interferon, IL-3 and the granulocyte macrophage colony stimulating factor and also is with much shorter survival without compromising its malignant cell killing efficiency 40, 41.

## Conclusions

CD4 is expressed in a significant proportion of AML patients. CD4CAR represents a novel approach to specifically and robustly target malignant CD4+ AML blasts. Effective remission induction or disease burden reduction is direly needed in preparation for allogeneic stem cell transplant in refractory AML patients. CD4CAR is expected to be effective and safe in this setting. Post-transplant engrafted T cells will rescue CD4 positive lymphodepletion after CD4CAR treatment and hence offset longer term immune suppression. Adoption of CAR technology in both T cells and NK cells provides versatility and greater therapeutic range for patients who previously had no options. CD4CAR T cell and NK cell therapy are effective methods for augmenting current AML therapies and their prompt application in clinical trials is warranted.

## Supplementary Material

Supplementary figures.

## Author Contribution

HS: Corresponding author. Participated in experimental design, analyzed data and wrote the paper. KP: second first author, designed, supervised and performed *in vivo* experiments and contributed to writing the paper. Masayuki Wada, Xiao Shuai, Lulu E. Yan, Jessica C. Petrov: Performed the experimental work. Yupo Ma: funded and supervised this work and senior author. All authors read and approved the final manuscript.

## Abbreviations

CD4CAR: CD4 Chimeric Antigen Receptor directed T cells
AML: Acute Myeloid Leukemia
HSCs: Hematopoietic Stem Cells
CAR-T cells: Chimeric Antigen Receptor T cells
NK: Natural killer
