# Supplementary figures and images for: Preclinical Targeting of Human Acute Myeloid Leukemia Using CD4-specific Chimeric Antigen Receptor (CAR) T Cells and NK Cells
Source: J Cancer. 2019 Jul 23;10(18):4408–19. doi: 10.7150/jca.28952 (PMC6691696; doi:10.7150/jca.28952)

**FIG. S1.**

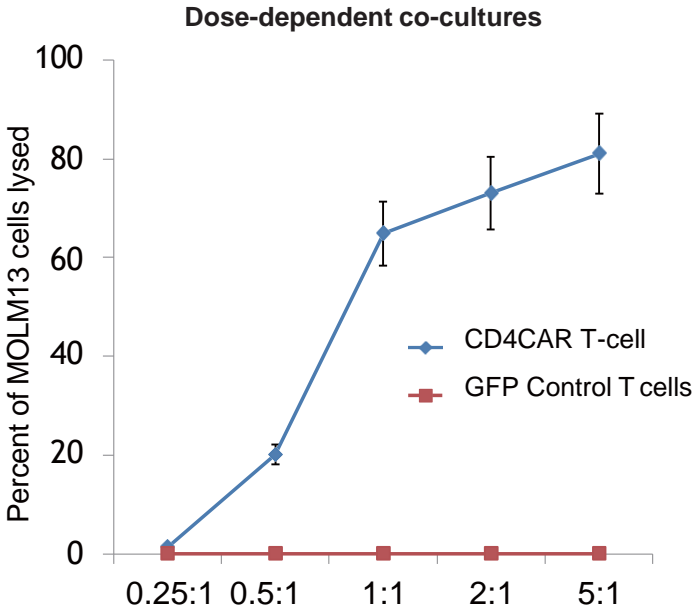

FIG. S2.

CAMPATH experiment design

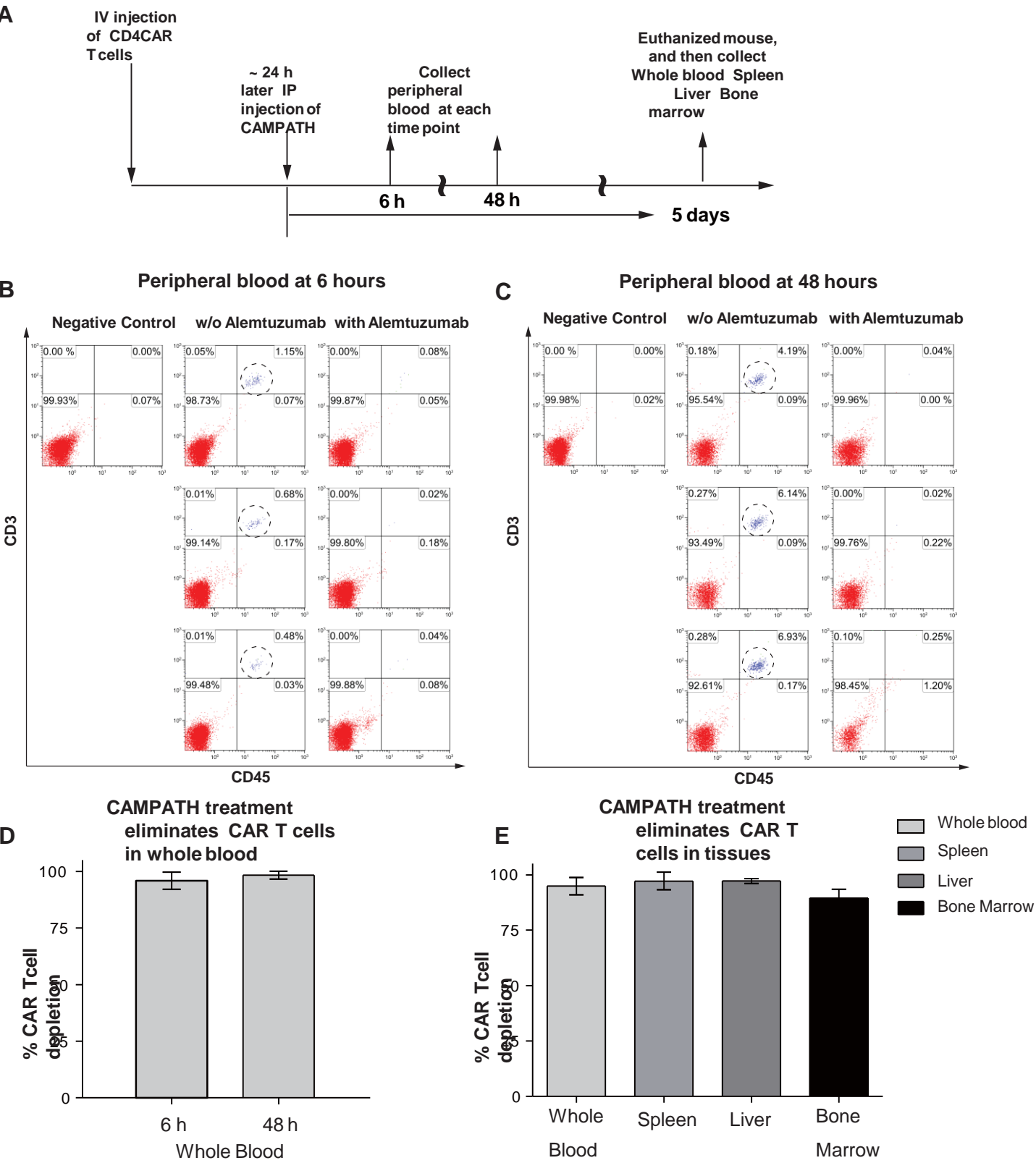

Supplement: Supplementary file 1 — Supplementary figures. [file jcav10p4408s1.pdf]
